# Role of spatial tools in public health policymaking of Bangladesh: opportunities and challenges

**DOI:** 10.1186/s41043-016-0045-1

**Published:** 2016-02-27

**Authors:** Dohyeong Kim, Malabika Sarker, Priyanka Vyas

**Affiliations:** 1 School of Economic, Political and Policy Sciences, University of Texas at Dallas, (GR 31), 800 W Campbell Road, Richardson, TX 75080-3021 USA; 2 James P Grant School of Public Health, BRAC University, Dhaka, Bangladesh

## Abstract

In spite of the increasing efforts to gather spatial data in developing countries, the use of maps is mostly for visualization of health indicators rather than informed decision-making. Various spatial tools can aid policymakers to allocate resources effectively, predict patterns in communicable or infectious diseases, and provide insights into geographical factors which are associated with utilization or adequacy of health services. In Bangladesh, the launch of District Health Information System 2, along with recent efforts to gather spatial data of facilities location, provides an interesting opportunity to study the current landscape and the potential barriers in advancing the use of spatial tools for informed decision making. This study assessed the current level of map usage and spatial tools for health sector planning in Bangladesh, focusing on investigating why map usage and spatial tools remained at a basic level for the purpose of health policy. The study design involved in-depth interviews, followed by an expert survey (*n* = 39) obtained through snowball sampling.

Our survey revealed that assessing areas with shortage of community health workers emerged as the top most for basic map usage or primarily for visualization purpose, while planning for emergency and obstetric care services, and disease mapping was the most frequent category for intermediate and advanced map usage, respectively. Furthermore, we found lack of inter-institutional collaboration, lack of continuous availability of trained personnel, and lack of awareness on the use of geographic information system (GIS) as a decision-making tool as three most critical barriers in the current landscape. Our findings highlight the barriers in increasing the adoption of spatial tools for health policymaking and planning in Bangladesh.

## Introduction

Even though there have been increasing efforts towards gathering spatial data in Bangladesh, usage of maps is mostly for visualization and descriptive purposes of health data [1–3]. According to the 2014 Bangladesh Country Report on statistical and geospatial information system, the use of spatial tools for the purpose of health policy decision making seems fairly nascent [4]. Furthermore, in the context of Bangladesh, only a handful of published studies have employed a spatial statistical approach mostly for disease mapping [5, 6] and demonstrated initiatives towards applying geographic information system (GIS) and spatial tools outside the realm of academic research [1, 7]. The launch of District Health Information System 2 (DHIS2) along with recent efforts to gather spatial data by the Management Information Systems Wing under the Director General of Health Services in Bangladesh provides an interesting opportunity to study the current landscape on the application of spatial tools and potential barriers in scaling up the use of GIS-based technology for public health policymaking [8].

Spatial tools in the form of basic indices such as Moran’s I, Geary’s ratio, and local indices of spatial autocorrelation (LISA) have been widely used in identifying local clusters of infectious diseases or any outbreaks, identifying communities at a high risk of not receiving access to government services, and targeting appropriate interventions [9]. However, health-related applications of advanced spatial tools integrated with statistical and mathematical techniques, such as spatial interpolation, network and service area analysis, and geographically weighted regression, are still at their infancy in developing world despite their predictive capabilities [10–12]. A few studies that demonstrate the application of spatial tools for informed health decision-making in developing countries include: understanding accessibility of health services in Kenya [13], identifying underserved communities by the health facilities in Costa Rica [14], establishing a spatial decision support system for health planners to ensure provinces fulfill their mandates for health service provision in rural British Columbia [15], and analyzing incidence rate of pneumonia for a hospital in central Brazil [16]. According to the literature, a combination of factors delaying widespread adoption of spatial tools in developing countries include lack of available spatial and attribute data, lack of awareness on the use of GIS, lack of technical capacity, lack of political willingness, inter-governmental factors, and lack of sharing of data [17–19].

In view of the limited studies that have examined the potential application of spatial tools and the barriers in the adoption of these tools, specifically in the context of Bangladesh, this study investigated:

1. What is the current level of map usage in health sector vis-à-vis other sectors in Bangladesh?
2. Why is the use of spatial tools and maps limited for actual health planning purpose?
3. What are the barriers to adopting spatial tools for targeting health interventions?

## Materials and methods

This study was conducted during a 3-week period using a mixed methods approach. Initially, we conducted in-depth interviews with nine experts who either represented key government organizations, academics, or worked in leading non-profit organizations. These in-depth interviews also served as a pre-test for the paper-based survey, and the survey instrument was modified accordingly. Preliminary survey instrument was approved by the Institutional Review Board office at University of Texas, Dallas. We targeted a purposeful sample of more than 100 respondents through snowballing technique to participate in the survey. In spite of this, only 39 surveys were completed and returned on time, with remaining either not given back in time or the respondents declining to participate. The high non-response rate was either due to lack of permission or support from the higher authorities within their organization to respond to the survey, or in some cases due to respondents’ own lack of knowledge and interest in the application of GIS for policy and planning. Among the participants who completed the survey, about 48.7 % were from government health agencies or other government departments that collect and maintain GIS data. Another 30.7 % of the respondents were from the major research organizations in Bangladesh, 12.8 % were from academic institutions, and 7 % of the returned survey did not have all the necessary demographic information filled. This being a key informant survey, it was mostly circulated among top-, middle-ranking officials, and others who were referred to by their peers or senior officers.

The survey had three components. The first section assessed respondent’s knowledge on the existence of maps and spatial data within their organization, along with the questions on the use of maps in the health sector vis-à-vis other sectors. In the next section, questions pertained specifically towards the use of maps in the health sector. To assess the level of map usage in the health sector, we classified map usage as “basic” if maps were created only for visualization and descriptive purposes and as “intermediate” if it was based on exploratory spatial data analysis (ESDA)—a method of extracting spatial pattern by combining multiple sources of data. It was viewed as “advanced” if it was based on a statistical model-based approach. The last section of the survey asked about the potential barriers in the adoption of spatial tools for the health field.

## Results

Our survey found that about 78 % of the respondents had geo-referenced maps or any other kind of spatial data available in their organization. The spatial data include administrative boundaries at different scales, road network, river network, and any form of spatial data. Approximately 28 % of the respondents stated that they had geo-referenced maps at the Upazilla level or the second administrative layer, while 26 % indicated they had spatial layers available for all the administrative units, including wards or the lowest administrative unit. With regard to technical capacity within their organization for map creation, 55 % of the respondents indicated that their organization was able to create maps to meet their own requirements, 61 % stated that their organization used available maps, and 32 % stated that their organization sought help from an outside agency to create maps for them.

**Fig. 1:**
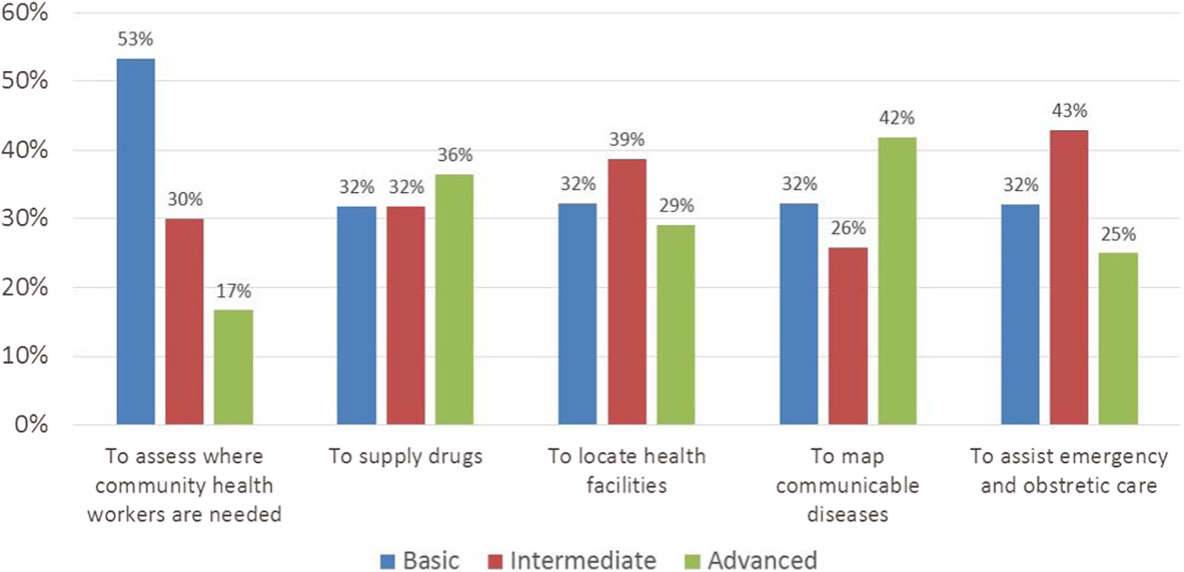
The use level of maps and spatial models by health policy agenda

Considering a high variability of map usage levels among the organizations depending on its purpose, the survey also investigated the level of GIS and spatial tools by the five categories of health policy agenda in Bangladesh, including identifying areas with the shortage of community health worker, allocating drug supplies, locating health facilities and service area, mapping communicable diseases, and assisting emergency and obstetric care. Figure 1 demonstrates the variation in the map usage within each of the categories of health policy and planning concerns. It is found that identifying areas with shortage of community health workers emerged as the top most category in which maps were used at a basic level mostly for visualization and descriptive purposes. The categories that were ranked the highest in intermediate map usage were assistance in emergency and obstetric services planning and allocation of drug supplies. In case of advanced map usage, disease mapping emerged at the forefront. No significant difference was found among the different types of organizations to which the respondents belong.

**Fig. 2:**
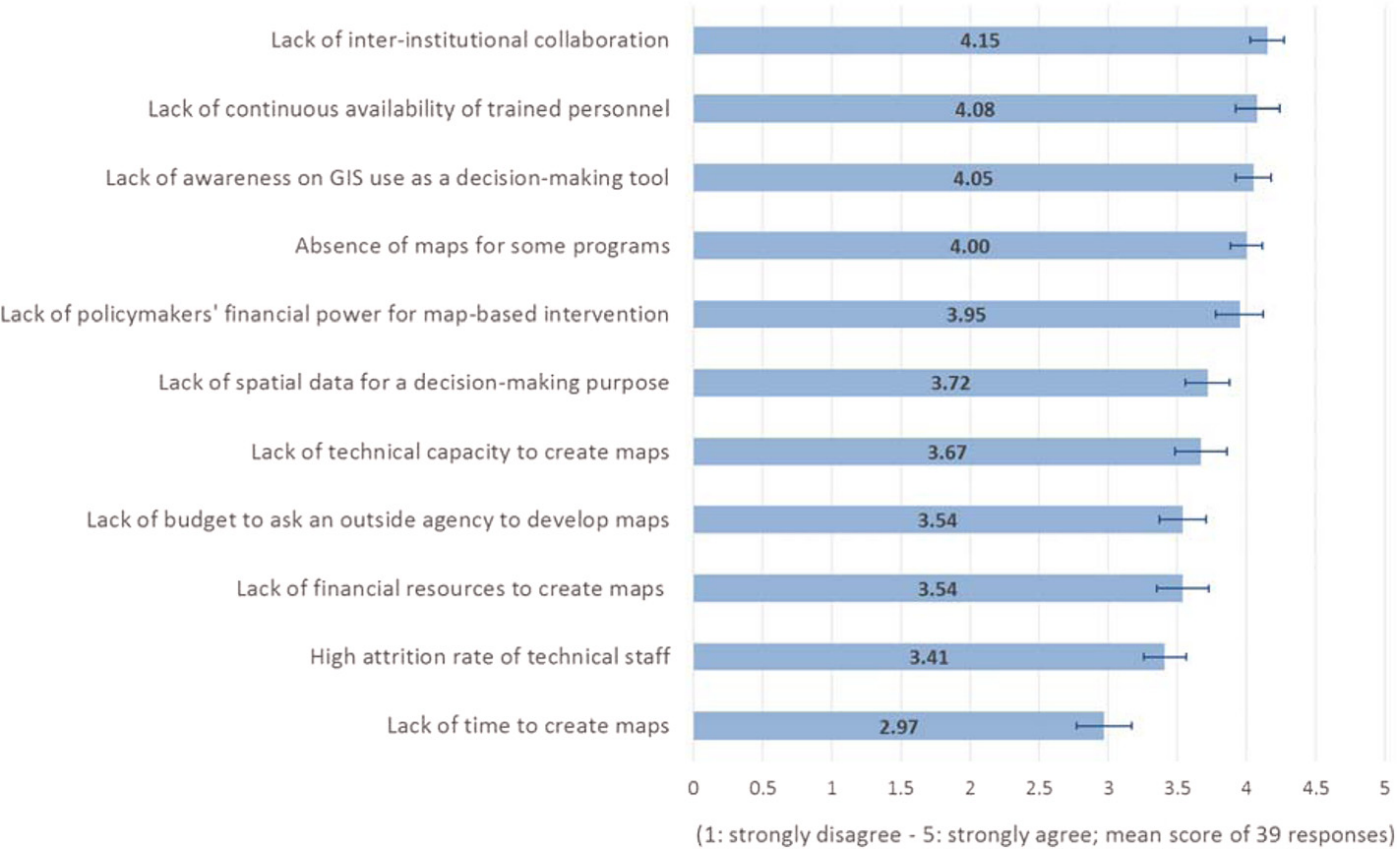
Barriers to using of maps as a decision-making tool for health intervention

It is interesting to note that, as shown in Fig. 1, there exist a significant level of advanced use of spatial tool across many health intervention sector, although uptake of these products for policymaking is limited. This gap is partially due to the fact that knowledge of advanced use of spatial tools is confined to and driven mostly by research-oriented scholars. In some instances such as communicable disease mapping, it is also driven by those working in multilateral organizations that work with the government closely. However, within the government agencies, knowledge of advanced spatial tools for decision-making purpose is limited, which was found to be one of the important barriers in Bangladesh, as shown in Fig. 2. Due to these factors, uptake in the application of advanced spatial tool for targeting health interventions or decision-making purposes has been limited.

Figure 2 reports the mean scores for 11 barriers towards the adoption of maps and spatial analysis as a decision-making tool for health intervention, along with standard error bars to show the level of variability of the scores among 39 respondents. The lack of inter-institutional collaboration was found to be the most important barrier mostly due to the lack of sharing of spatial data among different agencies. This has led to several agencies involved in generating the same kind of spatial data, ultimately resulting in duplication of efforts. The lack of continuous availability of trained personnel emerged as the second most important barrier. Some respondents felt that even though staff training was quite prevalent in Bangladesh, there was a high turnover of skilled staff to private high-paying jobs due to which knowledge was being lost and the process of map creation and spatial analysis was not sustained after few fragmented initiatives or efforts. In some cases, bureaucrats were assigned ad hoc roles and responsibilities without having any prior educational background or experience in that field, which in turn affected the level of acceptance and usage of these tools. The lack of awareness on the use of GIS and spatial analysis as a decision-making tool also emerged as a critical barrier because knowledge of spatial data creation and its utility in analyzing health problems was confined mostly to GIS teams in those organizations. In most cases, other people had very limited awareness on the use of maps in their organization or its capability in analyzing and evaluating health programs, which was observed by the researchers during the fieldwork. Besides, some respondents remarked that budgetary allocation and statistical analysis still continue to be in the domain of economists and statisticians, and sometimes they are not aware of the capability of the GIS-based tools to inform decision-making.

As also seen in Fig. 2, the lack of financial power to the district health managers was also one of the contributing factors due to which maps cannot be used for allocating financial resources as these decisions were mostly guided by political compulsions. Some respondents viewed the lack of spatial data as also a factor that made it less conducive to the use of spatial tools as several agencies are currently in the process of generating spatial data of various types and it would still take a few years before it can be linked to existing statistical data to conduct any analysis. The lack of technical capacity, lack of financial resources to outsource such tasks, lack of budget, attrition of technical staff, and lack of time were found to be the lowest on the list of barriers.

## Discussion

Given the current efforts undertaken by several government and non-profit organizations to gather spatial data in Bangladesh, this study highlights the opportunities available for performing spatial analysis to solve health policy problems. With increasing availability of street network data through initiatives such as the Open Street Map and Google Earth, existing efforts of gathering health facilities location data and other forms of spatial data provides an enhanced opportunity in the current context. However, several challenges lie ahead for policy makers to adopt these tools for decision-making purposes. Unless capacity building initiatives within government agencies and collaboration between different agencies for collecting and sharing GIS data improves, adoption of these tools on a regular basis for policymaking purposes may prove to be formidable challenge. By highlighting the barriers associated with the application of spatial tools such as those pertaining to the lack of continuous availability of trained personnel and the lack of awareness on GIS use as a decision-making tool, the study draws attention to the need to address those challenges. The findings from this study could inform a national tactical plan for GIS use in health analysis, including national guidelines for standard acquiring, processing, interoperability, and quality control of GIS data. Even though we do not intend to generalize our findings to other developing countries, we believe that some of the barriers noted in our study could have important lessons for other countries as well. Further studies for other countries in the Asia-Pacific region may gain from a comparative perspective on the challenges faced by developing countries in the adoption of spatial tools for policymaking in the health field.
